# “It's like a forgotten issue sometimes …”: Qualitative study of individuals living and caring for people with chronic breathlessness

**DOI:** 10.1111/crj.13652

**Published:** 2023-06-23

**Authors:** Anthony Sunjaya, Allison Martin, Clare Arnott, Guy Marks, Christine Jenkins

**Affiliations:** ^1^ Respiratory Division, The George Institute for Global Health UNSW Sydney Sydney New South Wales Australia; ^2^ Cardiovascular Division, The George Institute for Global Health UNSW Sydney Sydney New South Wales Australia; ^3^ Department of Respiratory Medicine, South Western Sydney Clinical School UNSW Sydney Sydney New South Wales Australia

**Keywords:** chronic obstructive, dyspnoea, heart failure, lung neoplasms, multimorbidity, patient‐centred care, pulmonary disease, self‐management

## Abstract

**Introduction:**

This study aims to explore the perspectives of patients and carers with chronic breathlessness on current provision of care, care expectations, and self‐management needs to develop relevant health services and resources to improve clinical outcomes.

**Methods:**

In‐depth semistructured interviews were conducted on patients living with chronic breathlessness and carers.

**Results:**

Thirteen patients (cardiac, respiratory, and noncardiorespiratory) and two carers were interviewed (mean age 57 years, 47% female, median duration with breathlessness 5 years). Four main themes were identified: (1) living with breathlessness, (2) diagnosis delays, misdiagnosis, and knowledge gaps, (3) beyond curing disease: symptom relief and improving quality of life, and (4) self‐management and limited support for it.

**Conclusion:**

Breathlessness has a high personal impact but remains a neglected condition in Australia. Patients suffer from lack of personal, community, and provider awareness, discontinuity of care, and too few clinical and self‐management options.

## Graphical abstract

A qualitative study of carers and patients with chronic breathlessness found patients suffer from lack of awareness, discontinuity of care, and too few clinical and self‐management options. To improve outcomes, community perspectives, practice, and system changes are required to improve identification, shorten time to diagnosis, provide wider treatment options especially nonpharmacologic support, and to empower patients to self‐manage.

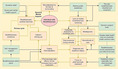

## 1 INTRODUCTION

Breathlessness is a common symptom associated with many prevalent diseases including chronic obstructive lung disease, asthma, heart failure, lung cancers, and post COVID‐19 syndrome.^1^ Previous qualitative studies^2^ have focused on exploring its impact, mostly in patients with chronic obstructive lung disease, rather than its impact as a symptom across a diverse set of causes.

Understanding the full range of patients' experience of living with breathlessness, their care expectations and self‐management needs are vital to develop relevant health services and resources to improve clinical outcomes as patients present with symptoms, rather than disease labels in practice. This study therefore aimed to describe the perspectives of patients and carers of patients with chronic breathlessness due to diverse causes, exploring living with breathlessness, receiving medical care, and accessing information regarding self‐management.

## 2 METHODS

This study is reported in accordance with the Standards for Reporting Qualitative Research.^3^ Further details are found in Table 1.

**TABLE 1:** Methods.

	Description
Ethical approval	University of New South Wales Human Research Ethics Committee (HC200534)
Design	In‐depth semistructured interviews (Interview Guide in Manuscript Supplement), each about 60 min long, undertaken virtually
Eligibility criteria	Adults (≥18 years old) who experienced chronic breathlessness (≥4 weeks) or are caring for someone with chronic breathlessness Patients or people who were being cared for should be ambulatory and had visited their general practitioner at least twice in the last 12 months as we sought to understand their recent experience in receiving medical care for breathlessness
Sampling	Participants were recruited based on a prespecified sampling frame to have representation across various genders, age groups, disease groups, states of residence, and settings (urban/rural) Participants were recruited until thematic saturation was reached and adequate diversity in participant profile was achieved
Recruitment	Recruitment was conducted online via two national patient registries
Topics explored	Experience of living with breathlessness Current medical care experience and their expectations Self‐management resources they use and need
Data collection	Conducted by the investigator (AS), a male medical graduate and PhD student with prior training and experience in conducting qualitative studies. Peer checking (process of checking a researcher's work by another person in that field) was conducted after the first three interviews between investigators (AS, CJ, and AM) before the remaining interviews were conducted. The interviews were recorded and transcribed verbatim
Compensation	Participants were provided gift vouchers post‐interview as compensation for their time
Analysis	Thematic analysis informed by the field notes generated during the interviews by AS was analysed using the software NVivo 12. The codes developed were then discussed with the other investigators (CJ, AM, CA, and GM) and difference in opinions were solved via consensus. Direct remarks from the participants are presented between quotation marks.

## 3 RESULTS

**FIGURE 1:**
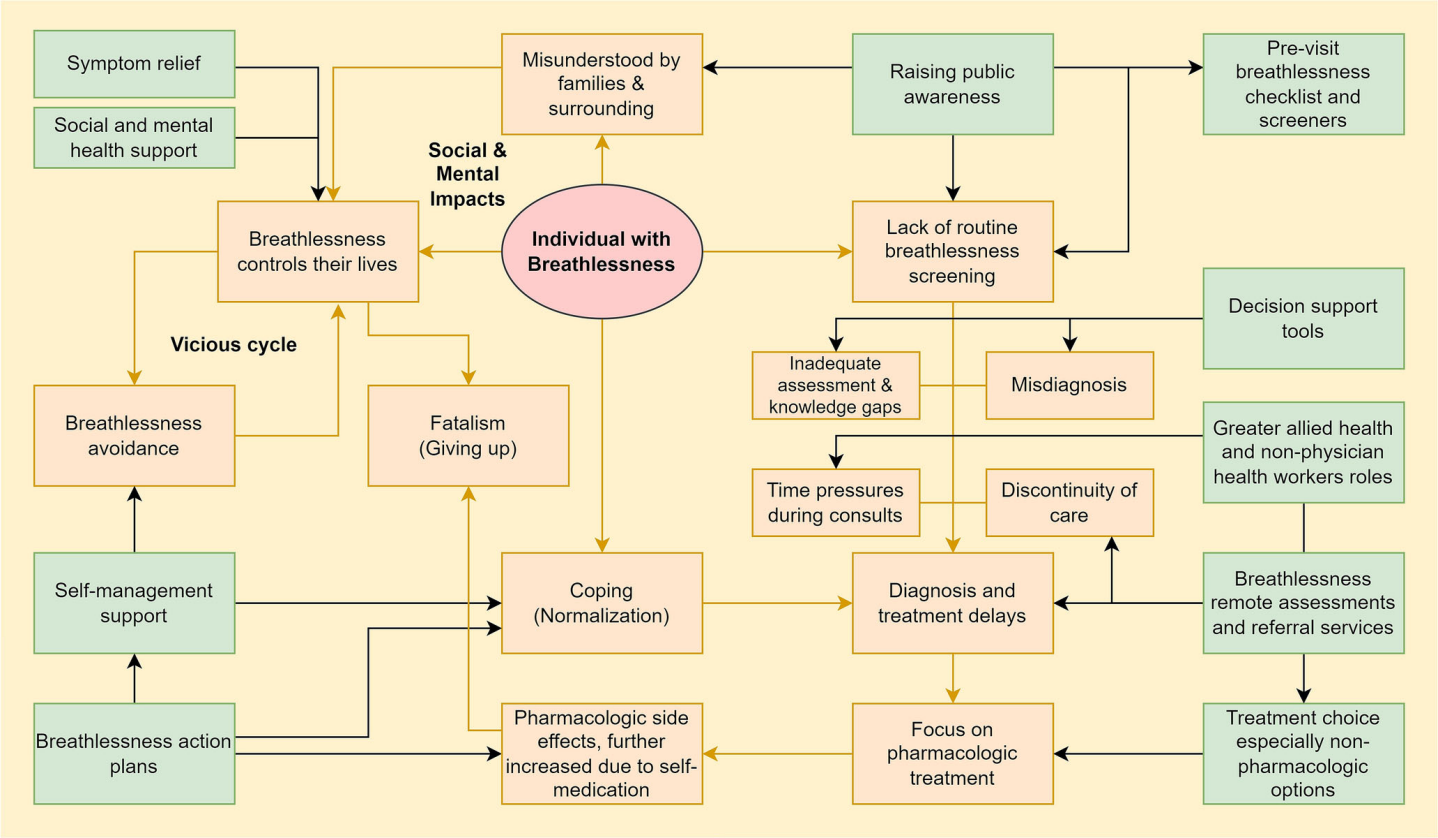
Summary of participants' identified gaps and their suggested solutions. Yellow lines show the linkages between the identified gaps, and black lines identify possible points of intervention for suggested solutions.

Fifteen individuals who had experienced breathlessness for a median of 5 years (Interquartile Range 2.75–9) were interviewed. Participants demographic profile can be found in Table S1. Four key themes were identified: (1) living with breathlessness, (2) diagnosis delays, misdiagnosis, and knowledge gaps, (3) beyond curing disease: symptom relief and improving quality of life, and (4) self‐management and limited support for it. Figure 1 summarises participant‐identified gaps in current care and their suggested solutions expanded below, with selected quotes presented in Table 2. Additional quotes are available in Table S2.

**TABLE 2:** Themes and selected quotes relating to them.

Theme/subtheme	Selected quotes relating to the theme
Theme 1—Living with breathlessness	
Breathlessness controls their lives and the vicious cycle	(I am) short of breath (when) doing anything including taking the bins out. (I need to) plan my day around stopping and breathing. (Participant 4, Female) (I've) got a 6‐year‐old son who wants me to get involved in sports, and I can't do a lot of that with him. (Participant 14, Male) Any amount of stress (and) I have a breathing problem straight away. (Participant 11, Male) The moment I try to do something I feel breathlessness … when I do something it's when it starts. A year ago, I can cook, now I can't even cook. Better to sit back and enjoy my breathing rather than get breathless. (Participant 15, Female)
Coping versus fatalism	Mentally OK, tend to put the situation at back of mind until somebody asks how I'm doing. Beyond that I have no great concern, I know the inevitable. (Participant 12, Male)
Theme 2—Diagnosis delays, misdiagnosis, and knowledge gaps	
Feeling misunderstood by those around them and by healthcare providers	One of my frustrations is that there was never a question on troubled breathing when you go for a health check‐up. I have doctors listen to my lungs but never ask me how's my breathing, how's my lungs. (Participant 5, Male) (During COVID‐19) When you have respiratory symptoms, they don't want you to come in, getting past the receptionist can be hard. (Participant 4, Female) Even walking from the waiting room to the consult room can be a problem (accessibility issues). (Participant 4, Female)
Discontinuity of care and knowledge gaps	It's hard to get the same doctor every time. It's hard to build rapport and need to explain it again and again even in the same practice. (Participant 8, Female) The sleep, lung and GP did not link, no one seem to be connected and investigate another area rather than their particular niche. (Participant 5, Male) Not sure how popular it is for people to come with this complaint, doesn't seem to be an avenue they can take to refer us for support, (the doctors) need to have some method that they can refer to …. (Participant 12, Male) Diagnosis is delayed sometimes by 2 years …. They (doctors) don't understand as they always change. (Participant 1, Female) Meeting a GP is very much on a time basis. (You meet for) a short while, (they) try to get you out and get the next one. Not much (time) to know more, (and) asking questions. (Participant 7, Male)
Theme 3—Beyond curing disease: Symptom relief and improving quality of life	
Clinician focus on pharmacologic management of breathlessness	Most doctor only give more Ventolin, (and no advice) to find out how I can improve it (my disease). (I would) love to know more about my disease. (Participant 7, Male)
Demand for choice, nonpharmacologic options, and support	I think every health organization should make diet and exercise as a tick able box to reduce the effects of any morbidity. Why don't we start with health and wellbeing? (Participant 5, Male) (I'm not) sure that they understand how severe it is, how (it is) physically impacting you, but mentally as well. (Participant 9, Male) What is actually happening, what's the prognosis. I do ask questions if I have the chance. (Participant 1, Female) Go out more, move more, (feeling) back alive. (Participant 9, Male)
Theme 4—Self‐management and limited support for it	
	(I have) lots of active hobbies, I want to have (my breathing) fixed so I can get back to what I want to do. (Participant 9, Male) (This is the) first time I've had an Asthma Action Plan (from their specialist). Now I can go (along the) medication ladder. Kind of makes me feel I'm more in control. Maybe the GPs can do that (more). (Participant 4, Female) Found out the Scleroderma Society of Victoria ‐ just found it other day. We are not even offered places to get (more) information, maybe most doctors think people just Google themselves. (Participant 5, Male) (Breathlessness) hasn't probably affected me so much that I get down that avenue. Love to read some peer reviewed articles to see my options. (Participant 7, Male) I deal with it relatively well pre‐emptively. I keep just purchasing the Ventolin on my own, as Flixotide need to get the script. Haven't been able to get a doctor. (Participant 7, Male) Have restarted oral prednisolone as I started to get more shortness of breath. (Participant 3, Male) I did Yoga for a while and used Yoga breathing techniques to help. It helps a lot. (Participant 8, Female) Resources on what to ask your GP (during a consult), you don't know what you don't know. (Participant 8, Female)

### 3.1 Living with breathlessness

#### 3.1.1 Breathlessness controls their lives and the vicious cycle

Participants provided various examples of what breathlessness felt like, how it controls their lives, and how routine activities can be a chore. They described how being breathless has impacted them socially and shared both physical and mental triggers for a breathlessness episode. For many, this has detrimentally affected their motivation to perform any physical activity which may result in worsening breathlessness. For some, breathlessness side effects from common medications and comorbidities such as “spine issues” interacted to further worsen activity avoidance.

#### 3.1.2 Coping versus fatalism

Participants shared a range of thoughts about breathlessness including normalising it, being content with their current state, and having a sense of accepting their inevitable demise due to their breathlessness. This fatalism compounded the poor health seeking behaviour of several participants who required external pressures from family or friends to seek help from health professionals.

### 3.2 Diagnosis delays, misdiagnosis, and knowledge gaps

#### 3.2.1 Feeling misunderstood by those around them and by healthcare providers

Families, friends, and the general public were reported to respond differently to breathlessness. When visiting their health providers, participants reported feeling that breathlessness was a “forgotten issue” and not raised during health check‐ups or their consultations. Participants however encouraged others to talk to their general practitioner (GP) regarding their breathlessness and emphasised the need for health provider education to raise breathlessness including having it as part of annual checks.

#### 3.2.2 Discontinuity of care

Participants reported poor continuity of care within and between specialties. Participants hypothesised this was due to the relatively rapid turnover of doctors, breaking down the doctor–patient relationship. They expressed mixed feelings on trusting the diagnosis from their health providers. Some had experienced substantial delays in getting assessed, while others felt they had been diagnosed too quickly, before a cause had been fully investigated. These situations were felt to be worse in the regional areas.

Participants acknowledged the time constraints in practice as a barrier to a complete assessment. However, some felt they were not adequately assessed in primary care even following multiple visits which they hypothesised may be due to a knowledge gap regarding diagnostic tests such as spirometry. Others appreciated the GPs doing the maximum that they can and encouraged people to find a doctor that listens and understands breathlessness. Even so, not all participants adhered to referrals for diagnostic purposes from their GP.

Participants raised the need for a clinical pathway for breathlessness for doctors, which would also include details of community‐based support services. They felt that the doctors were not adequately informed regarding where they and their patients can go for this information.

### 3.3 Beyond curing disease: Symptom relief and improving quality of life

#### 3.3.1 Clinician focus on pharmacologic management of breathlessness

Participants reported GPs' and specialists' tendency to focus on pharmacotherapy when managing breathlessness. They mentioned likely side effects and were interested to know what other medication options are available beyond what they have been prescribed.

#### 3.3.2 Demand for choice, nonpharmacologic options, and support

Participants wanted choice and support, especially for nonpharmacologic options that could improve their situation but felt they were not being offered. Many also conveyed the importance of improving their general health and well‐being via nutrition and physical activity advice, while acknowledging this may entail extra expense.

Participants were aware of the chronic and at times terminal nature of their condition. They accepted the “inevitable” but would like their doctors to focus on relieving their symptoms. They wanted doctors to go beyond the physical and show care about the mental impacts of breathlessness.

### 3.4 Self‐management and the limited support for it

All participants were keen to better understand their disease including practical ways to feel better. Participants believed being more aware of causes would enable them to do something about it. They also wanted access to someone to talk to about their disease, be educated about their disease, and provide a self‐management plan to help them feel more in control and functional. Most felt they did not receive adequate and relevant self‐management information and support but reported that learning from nonmedical sources, peers, and other patient bodies was beneficial.

## 4 DISCUSSION

Overall, the themes identified in this study suggest breathlessness remains a neglected condition in Australia. Patients suffer from lack of clinician awareness and expertise, poor community awareness, discontinuity of care, and too few clinical and self‐management options. This substantially impacts their clinical outcomes and quality of life. Problems and possible solutions evaluated in studies are described in Table 3.

**TABLE 3:** Summary of selected identified problems, solutions, and prior studies evaluating those solutions.

Problem	Possible solutions with selected references
Pre‐diagnosis of chronic breathlessness	
Lack of priority in elucidating this symptom by patients normalising it and clinicians focusing on physical examination with a stethoscope instead of asking about respiratory symptoms	Use of telehealth services to conduct a methodical respiratory assessment as was done during COVID‐19^4^ Develop standardised exertional tests to uncover “hidden” breathlessness in individuals who have reduced physically demanding activities to avoid breathlessness^5^
Stigma and misunderstanding of the importance of breathlessness in the community and among providers	Concerted effort to raise public awareness, prioritise assessment of breathlessness by including it in routine health check‐ups, and assisting affected individuals through checklists to prepare for their first breathlessness consultations Lowering the barrier to screening for breathlessness by digital resources such as the British Lung Foundation online breath test^6^
Diagnosis of chronic breathlessness	
Low utilisation of spirometry to aid diagnosis of chronic breathlessness‐related diseases	Community diagnostic centres where GPs can refer to Opening up direct referral to hospital‐based spirometry for GPs
Concerns of misdiagnosis by participants with rare conditions such as scleroderma and common ones such as asthma	Providing greater access to further diagnostic tests which are not always accessible in primary care as reported in prior studies with Australian GPs and specialists^7^, ^8^ Formation of breathlessness referral services^9^ to enable integrated assessment Provide primary care greater support, including digital resources and decision support systems^8^
Management of chronic breathlessness	
Lack of nonpharmacologic support as adjuncts to treatment	Previous studies have reported simple tools such as a hand‐held fan to relieve breathlessness^10^ Greater access to allied health and other nonphysician health professional deliver this support in addition to vital nonpharmacologic therapies such as pulmonary rehabilitation which significantly improve clinical outcomes^11^
Lack of action plans and patient education materials	Development of breathlessness action plans Development of high‐quality patient education materials and apps for breathlessness self‐management^12^

### 4.1 Strength and limitations

A strength of this study is the diverse group of participants undertaking interviews focused on the breathlessness experience rather than the causal disease, allowing thematic saturation to be reached despite the variety of underlying clinical causes. Limitations include the relatively small number of participants from minority ethnic communities and only two carers; hence, more studies are needed to fully understand their perspectives and needs. Furthermore, we were unable to include patients with breathlessness due to conditions such as neuromuscular disorders though a previous study^13^ in the United Kingdom have suggested similar experiences with regard to stigma, delays in diagnosis, and loss of independence.

## 5 CONCLUSION

Patients with chronic breathlessness suffer from lack of awareness, discontinuity of care, and too few clinical and self‐management options. To improve outcomes, community perspectives, practice, and system changes are required to improve identification of those with breathlessness, shorten the time to diagnosis, provide wider treatment options especially nonpharmacologic support, and to empower patients to self‐manage.

## AUTHOR CONTRIBUTIONS

**Anthony Sunjaya:** Conceptualization, Methodology, Data Collection, Investigation, Formal Analysis, Data Curation, Writing—Original Draft, Writing—Review and Editing, Visualization. **Allison Martin:** Project Administration, Formal Analysis, Writing—Review and Editing, Supervision. **Clare Arnott:** Writing—Review and Editing, Resources, Supervision. **Guy Marks:** Writing—Review and Editing, Supervision. **Christine Jenkins:** Conceptualization, Writing—Review and Editing, Resources, Supervision.

## CONFLICT OF INTEREST STATEMENT

All authors (AS, AM, CA, GM, and CJ) have no conflict of interest to disclose.

## ETHICS STATEMENT

Ethical approval for this study was obtained from the University of New South Wales Human Research Ethics Committee (HC200534).

## Supporting information


**Table S1.** Demographic characteristics of participants (n = 15).
**Table S2.** Themes and Sample Quotes. Number of participants contributing quotes to the sub‐themes are included within brackets.

## Data Availability

The data that support this study cannot be publicly shared due to ethical or privacy reasons and may be shared upon reasonable request to the corresponding author if appropriate.
