# Supplementary material for: “It's like a forgotten issue sometimes …”: Qualitative study of individuals living and caring for people with chronic breathlessness
Source: Clin Respir J. 2023 Jun 23;17(7):694–700. doi: 10.1111/crj.13652 (PMC10363784; doi:10.1111/crj.13652)
Supplement: Supplementary file 1 — Table S1. Demographic characteristics of participants (n = 15). Table S2. Themes and Sample Quotes. Number of participants contributing quotes to the sub‐themes are included within brackets. [file CRJ-17-694-s001.docx]

**MANUSCRIPT SUPPLEMENT**

“It's like a forgotten issue sometimes...”: Qualitative study of individuals living and caring for people with chronic breathlessness

Anthony Sunjaya^1^, Allison Martin^1^, Clare Arnott^2^, Guy Marks^3^ and Christine Jenkins^1^

*^1^Respiratory Division, The George Institute for Global Health, UNSW Sydney*

*^2^Cardiovascular Division, The George Institute for Global Health, UNSW Sydney*

*^3^Department of Respiratory Medicine, South Western Sydney Clinical School, UNSW Sydney*

**Corresponding authors:**

Dr. Anthony Sunjaya and Prof. Christine Jenkins, AM, MD, FRACP, FAHMS

Respiratory Division, The George Institute for Global Health, UNSW Sydney

5/1 King Street, Newtown, Sydney, Australia

Email: [asunjaya@georgeinstitute.org.au](mailto:asunjaya@georgeinstitute.org.au) / [christine.jenkins@sydney.edu.au](mailto:christine.jenkins@sydney.edu.au)

**Semi-Structured Interview Guide**

**Introduction:**

- Welcome and introductions.
- Confirm consent

**Topics and Questions:**

1. Have you or someone you care for experienced breathlessness?
2. What was the cause of your (or the person you care for) breathlessness? Can you share your (or their) experience living with it?
3. Have you (or the person you care for) sought help on assessing or treating your/their breathlessness? If yes, from whom?
4. Have you experienced any barriers or issues when you have received care for your (or the person you care for) breathlessness? What can be done better?
5. How would you describe the extent a GP would be able to handle your case? Which specialty would you think would be most relevant to help treat/diagnose your breathlessness?
6. What details would you want to have explained during a GP consult?
7. Are you (or the person you care for) using any self-management assistance or information to manage breathlessness at home? In medicine, we refer to supportive or assisting information as a “tool” and this can include education materials, videos, websites, and apps.
8. What information or assistance do you think would be most helpful in a self-management tool (education materials, website, app) for breathlessness?

**Table S1 – Demographic characteristics of participants (n=15)**

| Variable | N / Median (Min, Max) |
| --- | --- |
| Age (years old) | 57 (22, 84) |
| Sex |  |
| Male | 8 |
| Female | 7 |
| State |  |
| New South Wales | 3 |
| Western Australia | 5 |
| Victoria | 1 |
| Queensland | 3 |
| South Australia | 2 |
| Northern Territory | 1 |
| Disease Group (can be more than 1, except for No medical diagnosis and Carers) |  |
| COPD | 5 |
| Asthma | 6 |
| Heart Failure | 3 |
| Lung Cancer | 3 |
| Lung Infection | 2 |
| Interstitial Lung Disease | 1 |
| Ischaemic/Coronary Heart Disease | 2 |
| Heart Arrhythmia | 2 |
| Anxiety | 5 |
| Depression | 5 |
| No medical diagnosis | 1 |
| Carers | 2 |
| Duration of breathlessness (in years) | 5 (2.75, 9) |
| mMRC grade on recruitment^a^ |  |
| 0 | 1 |
| 1 | 2 |
| 2 | 6 |
| 3 | 2 |
| 4 | 1 |
| Change in mMRC grade between screening (a few weeks before interview) and on the day of the interview^a^ |  |
| Same or lower mMRC grade | 7 |
| Higher mMRC grade | 5 |
| Multimorbidity |  |
| Yes | 10 |
| No | 5 |
| Smoking |  |
| Current | 2 |
| Former | 6 |
| Never | 7 |
| BMI Category (Based on WHO BMI Classification) |  |
| Overweight and Obese (≥25) | 10 |
| Normal (18.5-24.9) | 2 |
| Underweight (<18.5) | 1 |
| Don’t know their weight/height | 2 |
| Socioeconomic status (Higher = less disadvantaged)^a,b^ |  |
| Quintile 1 | 1 |
| Quintile 2 | 1 |
| Quintile 3 | 6 |
| Quintile 4 | 3 |
| Quintile 5 | 2 |
| Education |  |
| Did not complete high school | 2 |
| Completed high school (Year 11 or 12) | 3 |
| Certificate or diploma | 6 |
| University degree | 4 |
| Speaks a non-English language at home |  |
| Yes | 2 |
| No | 13 |

*^a^No details for carers*

*^b^Based on postcode mapped to Socio-Economic Indexes for Areas (SEIFA) Index of Relative Socio-Economic Advantage and Disadvantage (IRSAD) 2016 released by the Australian Bureau of Statistics*

**Table S2 - Themes and Sample Quotes. Number of participants contributing quotes to the sub-themes are included within brackets.**

| **Theme** | **Sample quotes relating to the theme** |
| --- | --- |
| **Theme 1 – Living with breathlessness** | |
| Breathlessness controls their lives and the vicious cycle (10 of 15 participants) | Constantly out of breath, got to think what I do. … Everything's a bit of a chore. Even daily chores around the house can be a chore. Even putting on the dishwater can need me to sit down and need oxygen.  Turning over in bed can feel like a need to get more air. Even walking out into the kitchen I can feel I am breathless.  I used to be a singing teacher. It (Breathlessness) has affected me on singing, used to do that a lot during high school – (now it is) difficult to hold a note due to the weak lungs.  My office likes to do group power walk - struggled to do those walks, make a reason of catching coffee to go back to the office because I can't catch my breath.  During summer I have a group of friends I walk with, and I walk differently to them. During winter I can't due to the cold air (triggering my breathlessness).  I get right out of breath when walking, a little bit of it there, I still can't do physical labour I'm out of breath instantly, it goes back to lung function, lung capacity.  Find it difficult to walk due to breathlessness, I hardly walk at this stage. I mow my own lawns, I need to mow a little bit for 10 minutes come sit down then go back for another mow, so do it over 2-3 days.  Prior to diagnosis I was extremely fit, work on a farm, did heavy work, no problem at all. Now I have difficulty going to the shopping center, carrying bags, can't walk long distances as my legs hurt and lose my breath.  Since having a desk job I get more exercise induced asthma. There were not many issues when I was active in university.  I can't walk far because of spine issues. (I’m now) very breathless after walking short distances - puffing when walking to the car even if it is close.  Recently got off methadone and moved to buprenorphine (monthly injection). Overnight my breathing got better by at least 50%. Methadone is a very strong opioid - suppress my breathing, no idea how big an impact on my life and breathing. Hospital at least every year during winter due to not being able to breath (while on methadone). |
| Coping vs Fatalism  (4 of 15 participants) | Thought it was normal when small to be short of breath.  Respiratory physician said to come back in 6 months but don't think it can improve more than the current state, happy with my life.  The main point which really needs to be emphasised is to keep it at the back of mind, you need to manage the mental approach to it. Can't worry every minute of the day on the future, you can't change it. No point in chewing over it every day.  Came back to Australia - decide its emphysema, didn't seek help as you cannot do anything about it, you're on your own. (5 years later) My wife asks me to ask the doctor regarding my dry cough which comes with breathlessness.  One of my frustrations it was never a question are you having trouble breathing when you go for a health check-up. I have doctors listen to my lungs but never ask me how's my breathing, how's my lungs. Always just prod my history of depression. There's a disconnect that will pick up someone with a lung condition by asking them. Don't recall if I have been asked if I have an issue with breathing. |
| **Theme 2 - Diagnosis delays, misdiagnosis, and knowledge gaps** | |
| Feeling misunderstood by those around them and by health care providers  (6 of 15 participants) | For most general public there's a lot of misunderstanding of asthma or breathlessness and how to control it unless you know someone who has it and communicate it.  Have friends who panic even when I'm experiencing just the mildest of symptoms.  You go to a 15 minute consult, from the people I work with - they don't get taken seriously especially if you're an old woman in the countryside.  There’s disconnect at the GP stage but when the conversation started, they referred me quickly.  *Importance of being open to GPs*  It is really important to be open with your GP on what is going on with your health. Overtime it can deteriorate and there are ways to reduce that deterioration.  I explain the breathlessness through other pertinent disease to my GP, it can be quite dangerous if you don't take note of it.  There’s disconnect at the GP stage but when the conversation started, they referred me quickly. Once it was put up to him – he said we need to bring you to see a lung specialist. |
| Discontinuity of care and knowledge gaps (12 of 15 participants) | Diagnosis is delayed sometimes by 2 years; doctors are dependent on tests rather than brains and experience. (Participant who shared a story of a provider taking 2 years to find out cancer) Lots of people underdiagnosed for long times as they don't seem to see the same doctor. They don't understand as they always change.  The first doctor jumped the gun in diagnosing me with emphysema, it came right after I said I have smoked even after I tell him I have stopped. I was given the puffer, got serious side effects including diarrhea etc. Referred to the hospital for X-ray but cannot even walk up the stairs. Misdiagnosis in my view. Second doctor was good, meeting the doctor really made my day with his experience with lung issues in the UK. <Doctor’s name> has good understanding.  (My doctor) did a few quick checks then referred me to a lung specialist (6 month wait). After the initial consult I need to do some exercise, measure your breath, send for CT-scan, X-ray for my chest, bones and fingers. It’s pretty close to 4 years from emphysema then COPD then it became X then he did some blood tests then it became systemic sclerosis.  I have it (asthma) since I was a kid, diagnosed to have asthma but am not convinced it is the case.  Clot in lungs nobody picked that up, no one finds out. Disgraceful stuff, the medical system. Something’s seriously wrong in both hospital and doctors, they don't give the care they need. There are not enough doctors to come to the country. (You) don't get time to talk and then if you talk you will get a bigger bill when you meet on the table.  You think about House (Doctor in a TV show) thinking about every possibility under the sun, a 10 min consult doesn't allow anyone to do a particular exploration.  You get 10 mins if you are not a private patient, I have to remind myself on what I need to tell him. (Though) he's (GP) pretty good, (I) would want to spend more time with him being a patient with 3 chronic diseases.  GP - they've done what they can, frustrating and depressing at times not to be able to find the cause. The doctors are doing every test they can.  Beat the system - if you know your body, find a doctor that does listen. It's my body, I know my body. Never give up.  I have not touched upon this breathlessness issue to the GP. Waiting to speak to the oncologist.  The respiratory clinic follow up is really bad.  The doctor usually comes back and forth (temporary), (it’s) difficult to find a doctor. Relationship with the doctor has long gone. We didn't have a respiratory centre before but now there's one next town.  I never had a spirometry, he (GP) has never done that. It was helpful to go to a respiratory physician, without that I wouldn't know about the test. I had then asked my GP to send me for a lung function test. I need to push him to do the test and read the report.  There appears to be no knowledge is available on what support is available. I did ask earlier doctors on what help I can get but fell on deaf ears, didn't progress from there. There needs to be an information channel that they can go to.  Not sure how popular it is for people to come with this complaint, doesn't seem to be an avenue they can take to refer us for support, need to have some method that they can refer to if at all possible. Do it yourself from what I see, not getting any outside support. |
| **Theme 3 - Beyond curing disease: symptom relief and improving quality of life** | |
| Focus on pharmacologic management of breathlessness  (3 of 15 participants) | I bring it up to the GP, response depends on the GP - some of them are very quick to write a script to it because of the prior diagnosis, - not many ask further regarding it.  (Regarding GPs role) I think they can do the lung test and prescriptions, at that point not much more they can do.  The medical profession had no alternative offerings in their advice or armoury. They went sent straight to pharmacotherapy.  Lung specialists was very quick to go down the drug avenue while the side effects were horrendous.  GPs don't seem to understand bronchiectasis, Iranian doctor understood it. Aboriginal people getting it. Depends on the doctor, if they don't understand you need antibiotics, (you) need to fight for it and need to tell them. I'm type 1 diabetic, prednisolone makes it all worse |
| Demand for choice, non-pharmacologic options and support (10 of 15 participants) | Respiratory physician said to come back in 6 months, but I don't think it can improve more than the current state. (I’m) happy with my life. (I’d) like to have a voice, like not to get a tickle when I talk too much.  Rejected chemotherapy, prefer more quality of life than the negative effects of chemotherapy. Wouldn't feel chemotherapy will extent my life too much.  I have always read and been told that you can't improve your lung function but you can improve your lung capacity for a while I was consciously doing breathing exercises…. I used to find that help, it would be interesting to see what the spirometry would say  I want some support to help reduce the weight not just told to f**king lose the weight. I personally feel that if I can try losing 20 kilograms, some of my problems would go away.  Scared with the number of drugs that was put on the table, had a thought and another consult then shared my thoughts (with the GP). It was 3 visits before I decide I don’t want to go down the drug path. …. Trying to lose weight currently 90 kg, decided not to go down the steroids medication but focusing on lifestyle.  Is there anything I can do to help outside the Ventolin? Now I just (flow) anecdotal evidence from my experience, I don't know the science behind it. For example, why does swimming long term makes me feel better than running.  Exercise helps or not? Not sure about it - because you see one specialist and another then no one connects with each other, it's not like someone gets the (whole) facts.  (I) probably need to go to a physiotherapist – some of the barrier is financial, (as it is) costly.  <Participants’s name> this is what you have (disease name), from here I see 2 tracks the naturopaths (non-pharmacologic) route and a standard route of pharmacologic products then explain what the drugs do. Provide choice with regards to treatment, offer a range. (For example) can we do progressive dosing from smaller doses?  I’ve lost about 10 kilograms the last 3 months - it helped a lot.  No nutrition advice was provided just that I need to reduce my weight to 85 kg, no advice on to go to a particular service, exercise etc. Everything like hyperbaric, nutrition is all I look for from Dr Google.  I want to know what's available, probably a referral to a specialist  I would like to get a PT, I had a dietician. |
| **Theme 4 - Self-management and limited support for it.** | |
| (12 of 15 participants) | What is actually happening, what's the prognosis. I do ask questions if I have the chance. How the medicine works, what it's doing (one affects my throat, preach every Sunday, did a funeral recently though I was upset because my voice was croaky).  Just writing down the difference between my lungs and another who don't have asthma.  I think it's a good idea to go through if you are experiencing breathless for the GP to explain Ventolin, spacers, walk you through the steps, what to do if you didn't experience improvement, what to do if you have a heightened episode.  (For GPs) Explain what's happening and why certain things are being said by the specialist which was really informative  The practical stuff - wording like ILD, COPD all these acronyms are of no interest.  More things like this to discuss and talk to someone on what you can do to help, find ways to each state university (to participate in) clinical trials, (which are currently) hard to get in.  (I’m not) sure that they understand how severe it is, how (it is) physically impacting you, but mentally as well. A lot of doctors they don't care, they're in too much of a hurry, thankfully I haven't encountered too many of them.  Setup an Action Plan - I have one, most of the people I spent time with do not.  I look things up, read about all the drugs given online, find out how it's supposed to act etc.  I have gone and have a look myself on how to use spacers properly.  Every now and again I do read, through from no particular websites (Googling).  Scared when I visit Dr. Google.  I Google for government websites, official information, Asthma Australia website.  I have a look on Google, not overly driven by them more curiosity  Found out the Scleroderma Society of Victoria - just found it other day. See it's not even offered about place to get information, maybe most doctor think people just Google themselves. Didn't know if there's any organisations as I was told it was a rare thing.  I do keep a track if I've experienced some really high asthma days, if I find I've been sleepy, low PEFR. I make a note in my whiteboard, notes app or piece of paper, or text my Mum.  I did Yoga for a while and use Yoga breathing techniques to help. It helps a lot. Being taught to place my hands up my head to help my breathing problem. Walking around with my hands up.  (Been part of) COPD Facebook groups, done some tutorials and videos.  The Lung Foundation was helpful – (provided a) short, yearly ring.  Go to Asthma foundation Darwin to get a spacer which helped with Ventolin etc.  *Resources they would value*  Tips for when you are having breathing problems.  (List of) resources available to you if you want to follow up further - if you could list in your local area which doctors have a special interest in it, or work in the space.  Other people's experiences (with the same diagnosis as myself), what others have done that have benefitted themselves, most people I know have gone down the pharmacologic avenue but one shared how she had needed to stop in 6 months as she felt no benefit. The conversation with her was one of the lynchpins to go non-pharmacologic route.  A tracker that would help with Peak Flow, FEV1, FVC ratio (maybe as part of) an app. Something that would record symptoms, this is how breathless I'm today and what makes me breathless, trying to figure out the causation. Help you nut out what makes it worse so that you can prevent it.  Medication reminders and fitness program to improve deconditioning.  Info on how not only to deal with breathlessness, how to avoid being breathless e.g. areas with fumes etc cause breathless but not aware of other things than affect it such as air quality, dust etc  Videos, or clips on how to do things, educational videos.  I think it would be really nice if all the information is all in one place, a sort of patient guide, common symptoms, how a severe episode might look like, especially for those with not a lot of experience with asthma. This will be very beneficial to a lot of people, also encourage people to manage their symptoms better such as a few friends who do not manage their asthma really well. |
